# Interstitial Granulomatous Drug Reaction to Ustekinumab

**DOI:** 10.1155/2022/1461145

**Published:** 2022-03-25

**Authors:** A. Walker, J. S. Westerdahl, J. Zussman, J. Mathis

**Affiliations:** ^1^University of Utah School of Medicine, Salt Lake City, Utah 84132, USA; ^2^University of Utah, Department of Dermatology, Salt Lake City, Utah 84111, USA

## Abstract

Interstitial granulomatous drug reaction (IGDR) is a distinct inflammatory process that often presents as annular, violaceous plaques distributed on the extremities, proximal trunk, and intertriginous areas. The list of drugs implicated for inciting IGDR is growing, but most prominently includes ACE inhibitors, antihistamines, beta-blockers, antidepressants, and anticonvulsants. Ustekinumab is a human monoclonal antibody that targets inflammatory cytokines IL-12 and IL-23 and has been have shown to be effective in treating IGDR. However, we present a case that suggests ustekinumab can also act as an inciting agent for IGDR.

## 1. Introduction

Interstitial granulomatous drug reaction (IGDR) is a distinct inflammatory process characterized histologically by interstitial histiocytes distributed between reticular collagen bundles, prominent eosinophils, and lymphoid atypia [1]. It often presents as annular, violaceous plaques distributed on the extremities, proximal trunk, and intertriginous areas [2].

The list of drugs implicated for inciting IGDR is growing and currently includes ACE inhibitors, antihistamines, beta-blockers, antidepressants, and anticonvulsants alongside many others [1, 2]. Some biologics have been shown to be effective in the treatment of similar granulomatous processes including granuloma annulare and interstitial granulomatous dermatitis [3, 4]. Ustekinumab (Stelara), a human monoclonal antibody that targets inflammatory cytokines IL-12 and IL-23, is currently an effective treatment option for many chronic inflammatory conditions including ulcerative colitis (UC), Crohn's disease, psoriatic arthritis, and psoriasis, and case reports have suggested that it may also be effective in treating IGDR [3, 4]. However, we present a novel case that suggests ustekinumab can also act as an inciting agent for IGDR.

## 2. Case

**Figure 1:**
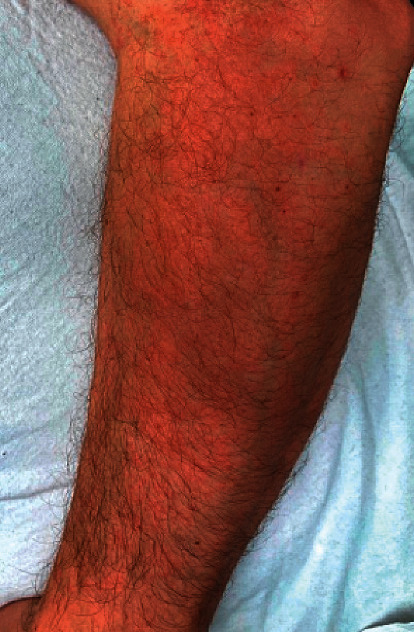
Pink papules coalescing into plaques on the left forearm. The rash extends into the posterior arm and is present bilaterally.

A 33-year-old male presented to our dermatology clinic for a pruritic rash. The rash was limited to his arms, but the pruritis involved his entire body. His medical history was significant for extensive biologic, steroid, and immunomodulatory therapy for the management of his UC. Three months prior, he switched from to facitinib to ustekinumab due to refractory symptoms. According to the patient, the rash presented shortly after the initiation of ustekinumab. Exam at that time revealed pruritic pink papules coalescing into plaques on bilateral forearms (Figure 1). Beyond that, he had been treated in the past with prednisone, budesonide, allopurinol, azathioprine, sulfasalazine, adalimumab, vedolizumab, and finally tofacitinib.

**Figure 2:**
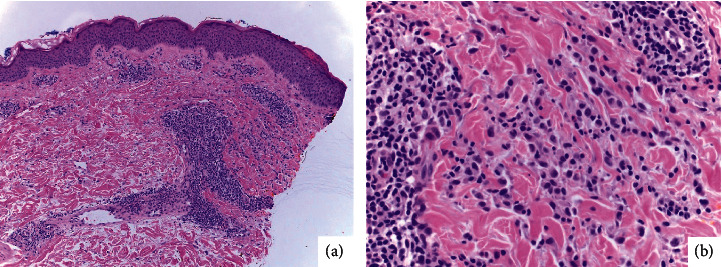
Histopathology with H&E demonstrating (a) epidermal acanthosis and mild spongiosis at 100X. There is dense perivascular inflammation, and interstitial histiocytes are noted at the edge of the specimen. (b) Histiocytes are seen between collagen bundles as well as scattered eosinophils, 400X.

A lesional punch biopsy was performed, which demonstrated spongiosis of the *epidermis*, perivascular lymphohistiocytic inflammation with scattered eosinophils, and interstitial histiocytes between superficial reticular collagen bundles (Figure 2). After biopsy, the patient did not improve with topical steroids and antihistamines. Instead, he elected to discontinue ustekinumab and restart vedolizumab with resolution of his symptoms. Excluding UC, the patient had no signs or symptoms of other autoimmune conditions, so further immunologic workup was not deemed necessary after cessation of ustekinumab resolved the patient's presenting complaint.

## 3. Discussion

We present a novel case of IGDR arising in a patient with ulcerative colitis after treatment initiation with ustekinumab. Based upon the clinical and histological findings, the patient was diagnosed as a probable case of drug reaction associated with ustekinumab, with a score of 5 (probable adverse drug reaction (ADR)) by the ADR Probability Scale [5]. No single clinical or histological feature is specific of drug reactions, and findings like spongiosis is common [6]. But the perivascular lymphohistiocytic inflammation and eosinophilia are consistent with interstitial granulomatous dermatitis and cannot otherwise be explained, particularly in the context of his medication history.

It is also possible for a granulomatous reaction to manifest as a part of the natural disease process of chronic inflammatory bowel disease (IBD) [7]. However, given the excellent control of underlying IBD on ustekinumab, onset after initiation of ustekinumab, resolution with its cessation, and lack of other possible etiologies at the time of onset, we believe ustekinumab to be the most likely culprit. Furthermore, interstitial granulomatous reactions caused by inflammatory bowel disease often have a neutrophilic component, which was not seen here.

The itching was bothersome enough that the patient wished to discontinue a medication that had improved his IBD significantly. Whether it could be “treated through” or would progress on the medication is not clear. Paradoxical adverse events (PAEs) related to biologic use are increasing in the literature and may complicate effective treatment of disabling diseases. PAEs include psoriasis, hidradenitis suppurativa, and other granulomatous disease processes linked to biologics, particularly anti-TNF inhibitor therapy [8]. We believe we have identified the first known case implicating ustekinumab as an inciting agent for IGDR.

## Data Availability

The patient data used to support the findings of this study are included within the article.
